# Case of Placenta Prævia—Central Presentation—Safe Delivery

**Published:** 1858-10

**Authors:** Richard M. Currey

**Affiliations:** Knoxville, Tenn., Professor of Medical Chemistry in Shelby Medical College, Nashville


*Case of Placenta Prævia—Central Presentation—Safe Delivery.* By Richard M. Currey, M. D., of Knoxville, Tenn., Professor of Medical Chemistry in Shelby Medical College, Nashville.

Early on the morning of th 12th of June, I was called to attend the lady of one of our citizens, who had during the night so frequently passed large clots of blood from the womb, that it filled her and attendants with alarm. It was yet two weeks to the expected time of her confinement, as she was the mother of seven children, and nothing of the kind had ever occurred in any of her previous confinements, she was fearfully apprehensive of some sad result to this labor. The first clot had passed off without any premonition having been given of its accumulation; and it had beeu so often repeated, that she had lost about a pint of blood. To my question, "Have you any pains, madam?" she replied, "none that could be called such, only that whenever the clots are expelled, there is a slight twinge in the womb, but it gives me no uneasiness." "Did you do any thing yesterday, madam, that could have produced a strain on the muscle of the bowels?" "Nothing at all that I can recollect, sir, except lifting a basket of fruit and placing it on the shelf." "Were your bowels moved during the day?" "Yes, sir, twice." "Was it a diarrhœa?" "No, sir."

Proposing an examination, I found the os tincæ high up in the cavity of the pelvis, and dilated to a very slight degree. Assuring her that labor had not as yet begun, I ordered the application of folded napkins to the abdomen, thoroughly wet with very cold water, administered an opium pill of one grain, and enjoined rest in the recumbent position, light diet and cold drinks.

At ten A. M. called, and found the discharge continuing every half hour, each clot averaging the size of a walnut. But examination revealed nothing more than in the morning. Continued cold cloths and rest.

At two P. M., sent for in haste; had just passed a large clot while attempting to rise in bed. Very dejected, and expressed her fears, through her tears, that she is to die this time.

On examination, the os tincæ was found dilated just sufficiently to admit the forefinger through it without any difficulty, when at once it came in contact with a surface rough and irregular, not smooth as the surface of the membranes. It also appeared to have a rawness about it, like torn flesh. Strengthened now in my belief that I had a case of placenta prævia to deal with, and assuring my patient as to the cause of the hœmorrhage, and that she should compose herself and allay her fears, as I would interfere in time to prevent any serious loss of blood, I ordered the application of cold ice-water to be constantly renewed, and that she should by no means raise her head from a level with the body. Finding no increase of discharge at the expiration of an hour, I withdrew.

At six P. M. called, and found that she had just begun to feel slight labor pains, occuring at lengthened intervals. On examination, found the mouth of the womb still further dilated, through which the rough raw, uterine surface of the placenta could be distinctly felt, and passing the finger round as far as it was prudent to do so, I became convinced that it was a case of *central placental presentation*. At each contraction, large clots of blood would be thrown off, showing the increasing danger of the case. Calling for strips of soft linen, I endeavored to introduce them as a tampon, but such was the rigidity of the external organs, and such pain was caused by them, that I was compelled to withdraw what had been introduced, and await the result.

A gradual dilatation took place in the womb, affording quite a convex surface to the presenting placenta; but now the blood, instead of coagulating, and being expelled in clots, came forth in a stream every time there was a pain.

Eight and a half P. M., a gush of blood came away, which caused the mother to believe the waters had been expelled. Believing that it was now my duty to interfere to save the life of the mother, as the discharge was very copious, and the pulse had begun to flag, I proceeded in this manner to effect my object:

At the time of a severe pain I pressed the finger firmly against the placenta, and gradually pushed, or rather worked it through the spongy mass till I reached the membranous surface. The penetration of this gave a free passage to the waters, and as the pain instantly subsided, I was enabled to ascertain the position and presentation of the child. This I found to be the first occipital presentation, though the head was still high up in the womb. Grasping the abdomen firmly in the left hand, snd making pressure upon it, I found the head descend at the return of a pain and began to engage in the expanded month. But, to my great fear, the placenta also pushed forward with it. The womb not seeming to act with as much energy, as I desired it should, I administered a full dose of powder of ergot, an article fresh and genuine, and upon which I could rely; a pain soon came on, but as it expelled the child, it continued to expel the placenta also, so that one half of it fully was now protruded in the vagina. This pain however was however succeeded by another, so quickly and so energetically, that my fears were allayed, for I found that it was acting so gradually and forcibly upon the child, as already to have passed it beyond the placenta, and, as it continued without cessation, it steadily expelled the child, delivery taking place one hour after the laceratioh of the placenta. The child was asphyxiated, and, as the cord was pulseless, it was detached; then by cold applications and friction, it in a short time gave signs of returning animation, and has done well.

The placenta was found lying loose in the vaginia, and on being removed was found lacerated within half an inch of the umbilical cord. The womb, by cold applications, contracted firmly, and both mother and child did as well afterwards as they could have done under the most favorable circumstances.

I have been thus minute in detailing the case, for the sole reason of saying that I believe its success depended *upon its being left alone after the presenting placenta was ruptured.* I do not believe that either of the other modes recommended, either turning or moving the placenta first, would have been any more successsul. I do not believe they would have been as much so. It rarely happens in cases of this character that both mother and child are saved, the one or the other almost always losing life. In the management of this case my first object was to introduce the tampon so as to facilitate labor, but in its introduction I failed. I did not use ergot earlier in the stage of the labor, because I did not wish to tear asunder the placenta from the uterine surface before it had presented so far in advance that when I should lacerate it, the child might pass through it without any delay; consequently I withheld its administration until it was necessary to use it to *expel the child,* and then in such quantity, that when it begun to act, *it should continue to do so till it had accomplished the object for which I administered it.* The plan pursued I regarded as the safest and best, and the result proved the wisdom of it. My whole course consisted simply in trying to hold the hemorrhage in check by cold applications and recumbent position, until the favorable time for the laceration of the spongy placenta, through which I hoped and was gratified in seeing, the child safely pass, aiding in its expulsion by one dose of ergot, and firm pressure upon the abdomen.

This was the first case of this character that has fallen to my charge in twenty years' practice, although several times I have noticed slight hemorrhages previous to delivery, doubtless owing to a partial implantation of the placenta, none of which required any interference whatever.—*Nashville Monthly Record.*

